# Hip-Spine and Knee-Spine Syndrome: Is Low Back Pain Improved After Total Hip and Knee Arthroplasty?

**DOI:** 10.7759/cureus.57765

**Published:** 2024-04-07

**Authors:** Vasileios A Kechagias, Theodoros B Grivas

**Affiliations:** 1 Orthopedics, General Hospital of Lemnos, Lemnos, GRC; 2 Orthopedics and Traumatology, Tzaneio Prefecture General Hospital of Piraeus, Piraeus, GRC

**Keywords:** total knee arthroplasty, total hip arthroplasty, low back pain, knee-spine syndrome, hip-spine syndrome

## Abstract

Introduction: Hip and knee osteoarthritis (OA) and low back pain (LBP) are prevalent diseases that can negatively impact daily activities. The concurrent existence of lumbar spine disorders with hip or knee issues forms two syndromes: hip-spine syndrome (HSS) and knee-spine syndrome (KSS). The primary objective of this study is to evaluate the relationship between hip and knee OA and LBP, as well as the changes to LBP after total hip arthroplasty (THA) and total knee arthroplasty (TKA). The secondary objective is to identify the cause of LBP among patients with hip and knee OA.

Material and methods: The group of hip OA patients treated with THA consisted of 34 individuals, and the group of knee OA patients treated with TKA consisted of 45 individuals. In these two groups of patients, the LBP was assessed using the visual analog scale score preoperatively and four and 12 months postoperatively. To determine the cause of LBP, we compared preoperative and postoperative (12-month) pelvic obliquity and hip or knee pain in patients with and without preoperative LBP.

Results: For hip OA-THA, more than half (55.88%) of patients suffering from hip OA also experienced moderate to severe LBP. Improvement in LBP was noticed in 79% of these patients at both four and 12 months post-THA, with levels dropping from 6.84 to 2.58 and then 2.53, respectively. Moreover, improvements in hip pain and pelvic obliquity were observed in patient groups both with and without preoperative LBP following THA. This suggests that there's no obvious correlation between LBP and these parameters.

For knee OA-TKA, most (62.22%) patients with knee OA experienced moderate to very severe LBP. In 50% of these patients, LBP showed improvement at four and 12 months post-TKA (6.39 → 4.79 → 4.04). Additionally, in both groups with and without preoperative LBP, knee pain and pelvic obliquity were improved after TKA, suggesting no clear association between LBP and these parameters.

Conclusions: HSS and KSS were frequently observed in patients. A significant improvement in LBP was seen after THA or TKA, suggesting that total arthroplasty should be prioritized before spinal surgery. Furthermore, there is not a definitive link between LBP, joint pain, and pelvic obliquity.

## Introduction

The prevalence of hip and knee osteoarthritis (OA), as well as low back pain (LBP), is on the rise due to the aging population.

Hip and knee OA can significantly hinder daily activities, including walking, ascending or descending stairs, and transitioning from sitting to standing. These conditions disproportionately affect the elderly, often leading to decreased mobility, a loss of independence, and an increased reliance on healthcare services. In the United States of America (USA), radiological hip and knee OA was found in approximately 28% of African-American and Caucasian-White individuals over 45 years old, with 10% showing symptomatic hip OA and 16% symptomatic knee OA. It is estimated that the risk of developing symptomatic hip OA approaches 25% by age 85, and for symptomatic knee OA, the risk approaches 50%.

LBP is defined as pain and stiffness localized below the costal margin and above the inferior gluteal folds, with or without leg pain. The cause of this pain, in most instances, can either be mechanical or arise from specific conditions such as inflammation, fracture, or malignancy. LBP is categorized as acute (if lasting less than six weeks), subacute (if lasting between six and 12 weeks), and chronic (when it exceeds 12 weeks). Every year, 10% to 30% of the adult population in the USA experiences LBP, with a lifetime occurrence reaching between 65% and 80%. Within the elderly demographic, the occurrence ranges between 21% and 75%. Globally, LBP was found to have a prevalence of 9.4%, making it the leading cause of disability and the sixth most significant overall burden. In the working-age population, it is notably the primary reason for disability, limited activity, and depression. The economic impact of LBP is significant, leading to early retirements and decreased productivity. In 2005, the USA alone spent over $100 billion on LPB-related healthcare.

Hip-spine syndrome (HSS) refers to the simultaneous occurrence of disorders in the lumbar spine and hip. This syndrome was initially characterized into four subcategories: simple, secondary, complex, and misdiagnosed HSS, by Offierski and MacNab in 1983 [1]. Wolfe et al. were the first to describe the co-occurrence of LBP and knee OA disorders [2]. Tsuji et al. later labeled the simultaneous manifestation of the lumbar spine and knee disorders as a knee-spine syndrome (KSS) [3]. Within the international literature, two primary theories exist to explain HSS and KSS. These suggest that LBP arises from either the anatomical irregularities of the trunk or from the pain induced by the hip and knee joints.

The primary objective of this study is to evaluate the correlation between hip and knee OA and LBP and observe changes in LBP following total hip arthroplasty (THA) and total knee arthroplasty (TKA). The secondary objective is to assess the correlation between LBP, joint pain, and pelvic obliquity.

## Materials and methods

This prospective study investigates the correlation between hip and knee OA and LBP. Additionally, it examines the impact of THA and TKA on LBP. The subjects were patients who underwent surgery for severe OA in their hip or knee between April 2014 and March 2016. Patients diagnosed as having radiographic evidence of severe unilateral hip or knee OA (Kellgren and Lawrence grades 3 or 4) were admitted to the study and assessed for inclusion. We defined certain exclusion criteria, including (1) significant OA in other lower extremity joints; (2) arthritis induced by other conditions, such as ankylosing spondylitis, rheumatoid arthritis, developmental dysplasia, and trauma; (3) neurological issues in lower extremities; (4) previous surgical procedures conducted on the spine or lower extremities; and (5) medical conditions affecting the body's alignment.

The group treated with THA for hip OA consisted of 34 patients, 15 men and 19 women, with a mean age of 67.62 ± 8.83 (range: 47-84 years). The mean weight of the patients was 82.32 ± 17.73 kg, the mean height was 165.00 ± 8.80 cm, and the mean body mass index (BMI) was 29.72 ± 4.31 kg/m^2^. Similarly, the group treated with TKA for knee OA contained 45 patients, 11 men and 34 women, with a mean age of 72.42 ± 6.98 (range: 54-90 years). The mean weight of the patients was 79.87 ± 13.79 kg, the mean height was 162.16 ± 5.89 cm, and the mean BMI was 30.36 ± 4.48 kg/m^2^. In these two groups of patients, the LBP was assessed using the visual analog scale (VAS) score preoperatively and four and 12 months postoperatively.

Our team's previous study, using the same patient sample, revealed abnormal trunk morphology in patients with hip and knee OA compared to normal subjects. One year after undergoing THA and TKA, only the patient's pelvic obliquity in the frontal plane showed improvement compared to preoperative values. The Design Institute for Emergency Relief Systems formetric four-dimensional analysis system was used to calculate the parameters of the trunk in all planes. This device is a surface topography instrument, and the final result is a high-resolution three-dimensional back shape reconstruction [4].

We evaluated the cause of LBP by comparing hip or knee pain and pelvic obliquity before surgery and 12 months after surgery in patients with and without LBP. The LBP and hip or knee pain were assessed using the VAS, while pelvic obliquity was measured in degrees.

This study received approval from Attikon University General Hospital's Institutional Review Board (approval number: ΕΒΔ390/9-9-2014, approval date: 24-9-2014) and consent from all participants. We conducted statistical analyses with SPSS Statistics version 17.0 (SPSS Inc. Released 2008. SPSS Statistics for Windows, Version 17.0. Chicago: SPSS Inc.), setting statistical significance at p≤0.05. The analysis of the variance model with one-way repeated measures (ANOVA) was used for a longitudinal study of LBP and comparisons using the Bonferroni test. The Wilcoxon test determined the correlation between LBP, hip or knee pain, and pelvic obliquity.

## Results

Hip OA, THA, and LBP

Approximately 55.88% of patients, 19 of 34, suffering from hip OA experienced moderate to very severe LBP, as evidenced by a VAS score of 3-10. In this patient group, the intensity of LBP was evaluated both before surgery and four and 12 months after the THA.

The findings are outlined in Table 1. A significant statistical and clinical difference was observed in the time measurements for LBP (p<0.001). Pairwise comparisons revealed substantial differences between preoperative measurements and those taken four months (6.84 to 2.58, p<0.001) and 12 months (6.84 to 2.53, p<0.001) after the operation.

**Table 1:** Longitudinal change of LBP in patients with hip OA before and after THA LBP: low back pain, OA: osteoarthritis, THA: total hip arthroplasty

	LBP		Pairwise comparison	
	Mean value	Standard deviation	4 months postoperative	12 months postoperative
Preoperative	6.84	2.09	<0.001	<0.001
4 months postoperative	2.58	2.17	-	0.992
12 months postoperative	2.53	2.22	-	-
p-value	<0.001			

The postoperative change in LBP volume was an intriguing measurement. Twenty-one percent of patients retained the same LBP score even four and 12 months after surgery. Conversely, 79% of patients saw their LBP score reduce, with 47.4% experiencing a significant decrease of more than four units at both the four- and 12-month postoperative markers. These results are compiled in Table 2.

**Table 2:** Longitudinal change of LBP in patients with hip OA before and after THA LBP: low back pain, OA: osteoarthritis, THA: total hip arthroplasty

Change in LBP volume	Change at 4 months postoperative		Change at 12 months postoperative	
	Number of patients	% of total patients	Number of patients	% of total patients
-9	-	-	1	5.3
-8	4	21.1	3	15.8
-7	2	10.5	2	10.5
-6	2	10.5	2	10.5
-5	1	5.3	1	5.3
-4	2	10.5	2	10.5
-3	3	15.8	3	15.8
-2	-	-	-	-
-1	1	5.3	1	5.3
0	4	21.1	4	21.1

To assess the correlation between LBP, hip pain, and pelvic obliquity, we conducted a study on two groups of patients. One group consisted of patients without LBP (n=15), whereas the other included patients with LBP (n=19) before surgery.

The results for patients without preoperative LBP are shown in Table 3. There was a significant reduction in hip pain from preoperative levels to 12 months post-surgery (p=0.001), with the mean hip pain score dropping from 8.67 to 2.27 and the median decreasing from 9.0 to 1.0. Similarly, pelvic obliquity also showed a statistically significant decrease over the same period (p=0.031), with mean levels falling from 5.53 to 2.93 and median values decreasing from 5.0 to 2.0.

**Table 3:** Longitudinal change of hip pain and pelvic obliquity before and after THA in patients without LBP preoperatively THA: total hip arthroplasty, LBP: low back pain, IQR: interquartile range

	Hip pain			Pelvic obliquity		
	Mean	Median	IQR	Mean	Median	IQR
Preoperative	8.67	9.0	1.0	5.53	5.0	6.0
12 months postoperative	2.27	1.0	2.0	2.93	2.0	5.0
p-value	0.001			0.031		

The outcomes for the group of patients who experienced LBP prior to surgery are outlined in Table 4. There was a significant decrease in hip pain from before the surgery to 12 months afterward (p<0.001). More precisely, the levels of hip pain were notably lower 12 months post-surgery (mean = 1.05, median = 1.0, IQR = 2.0) compared to the pre-surgical measurements (mean = 8.95, median = 9.0, IQR = 0.0). Pelvic obliquity also saw a significant change 12 months post-surgery (p=0.040) compared to preoperative status. At 12 months post-surgery, obliquity was notably lower, with a mean value of 2.05, a median of 2.0, and an interquartile range (IQR) of 3.0 as compared to the mean value of 3.26, a median of 2.0, and an IQR of 5.0 before the operation.

**Table 4:** Longitudinal change of hip pain and pelvic obliquity before and after THA in patients with LBP preoperatively THA: total hip arthroplasty, LBP: low back pain, IQR: interquartile range

	Hip pain			Pelvic obliquity		
	Mean	Median	IQR	Mean	Median	IQR
Preoperative	8.95	9.0	0.0	3.26	2.0	5.0
12 months postoperative	1.05	1.0	2.0	2.05	2.0	3.0
p-value	<0.001			0.040		

Patients in both groups showed comparable changes in hip pain and pelvic obliquity after THA. These results indicate that there is no clear association between LBP and hip pain or pelvic obliquity.

Knee OA, TKA, and LBP

In our study, we found that 62.22% of knee OA patients, 28 out of 45, reported moderate to severe LBP, with a score of 3-10 on the VAS. We monitored this group's LBP volume pre-surgery as well as four and 12 months post-TKA.

The results are outlined in Table 5, showing a statistically and clinically significant difference in the time measurements for the LBP (p<0.001). Pairwise comparisons reveal noticeable differences between preoperative measurements and those at four months (from 6.39 → to 4.79, p=0.001) and 12 months (from 6.39 → to 4.04, p<0.001) postoperation. A significant change was also observed between the measurements from the fourth to the twelfth month (from 4.79 → to 4.04, p=0.05).

**Table 5:** Longitudinal change of LBP in patients with knee OA before and after TKA LBP: low back pain, OA: osteoarthritis, TKA: total knee arthroplasty

	LBP		Pairwise comparisons	
	Mean value	Standard deviation	4 months postoperative	12 months postoperative
Preoperative	6.39	2.06	0.001	<0.001
4 months postoperative	4.79	2.10	-	0.050
12 months postoperative	4.04	2.49	-	-
p-value	<0.001			

The change in LBP volume postoperatively was an interesting measure to note. Approximately half of the patients maintained the same LBP score at the preoperative stage and four and 12 months postoperatively. Meanwhile, 50% of patients showed a decrease in the LBP score. A significant decrease of more than four units at four months was observed in 10.7% of patients, while 28.6% had a significant decrease in LBP at 12 months postoperatively. The results are collated in Table 6.

**Table 6:** Longitudinal change of LBP in patients with knee OA before and after TKA LPB: low back pain, OA: osteoarthritis, TKA: total knee arthroplasty

Change in LBP volume	Change at 4 months postoperative		Change at 12 months postoperative	
	Number of patients	% of total patients	Number of patients	% of total patients
-9	-	-	-	-
-8	-	-	1	3.6
-7	-	-	2	7.1
-6	1	3.6	-	-
-5	2	7.1	5	17.9
-4	3	10.7	3	10.7
-3	4	14.3	2	7.1
-2	2	7.1	-	-
-1	1	3.6	1	3.6
0	15	53.6	14	50.0

We assessed the correlation between LBP, knee pain, and pelvic obliquity by dividing patients into two groups. The first group consisted of patients without LBP (n=17) and the second comprised patients with preoperative LBP (n=28).

The results for patients without preoperative LBP are compiled in Table 7. A significant statistical difference in knee pain was observed from the preoperative phase to 12 months postoperation (p<0.001). Specifically, knee pain was significantly reduced 12 months after the operation, with a mean score of 1.53 and a median score of 1.0, as compared to the preoperative scores (mean = 8.53, median = 9.0). Similarly, pelvic obliquity also showed a significant statistical change from preoperative to 12 months postoperation (p=0.014). The mean score declined to 2.06, and the median score remained consistent at 2.0 after the operation, as compared to preoperative measures.

**Table 7:** Longitudinal change of knee pain and pelvic obliquity before and after TKA in patients without LBP preoperatively TKA: total knee arthroplasty, LBP: low back pain, IQR: interquartile range

	Knee pain			Pelvic obliquity		
	Mean	Median	IQR	Mean	Median	IQR
Preoperative	8.53	9.0	2.0	3.41	2.0	4.0
12 months postoperative	1.53	1.0	3.0	2.06	2.0	4.0
p-value	<0.001			0.014		

The results for preoperative LBP patients are detailed in Table 8. There was a statistically significant decrease in knee pain from preoperative to 12 months postoperative (p<0.001), with the pain levels notably lower at 12 months (mean = 2.43, median = 1.0, IQR = 2.0) compared to preoperative measurements (mean = 8.79, median = 9.0, IQR = 0.0). Pelvic obliquity also showed a significant improvement from preoperative to 12 months postoperative (p=0.002). Specifically, at 12 months, pelvic obliquity was lower (mean = 1.71, median = 1.5, IQR = 3.0) than preoperative measurements (mean = 3.18, median = 2.0, IQR = 3.0).

**Table 8:** Longitudinal change of knee pain and pelvic obliquity before and after TKA in patients with LBP preoperatively TKA: total knee arthroplasty, LBP: low back pain, IQR: interquartile range

	Knee pain			Pelvic obliquity		
	Mean	Median	IQR	Mean	Median	IQR
Preoperative	8.79	9.0	0.0	3.18	2.0	3.0
12 months postoperative	2.43	1.0	2.0	1.71	1.5	3.0
p-value	<0.001			0.002		

Both patient groups demonstrated similar alterations in knee pain and pelvic obliquity after TKA. These findings suggest a lack of substantial correlation between LBP and these parameters.

## Discussion

Hip OA, THA, and LBP

In this prospective study, we found that 55.88% of patients with severe hip OA who underwent THA also experienced moderate to severe LBP. There was a significant decrease in the VAS score for LBP in these patients, from 6.84 preoperatively to 2.58 four months postoperatively, and finally to 2.53 at 12 months postoperatively. Remarkably, the LBP scores dropped for 79% of the patients. Moreover, 47.4% of patients experienced decreases of more than four units in their LBP scores at both four and 12 months postoperatively.

These results align with those from similar studies. Vigdorchik et al. noted that 41% of patients with hip OA experienced LBP before THA, with LBP resolving in 82.4% and 91.2% of patients at the one- and two-year follow-up, respectively [5]. Weng et al. revealed that 56.5% of hip OA patients had LBP before surgery, with 17 reporting complete relief and 21 experiencing significant LBP relief after one year [6]. The average VAS score for LBP declined from 4.6 to 1.5 post-THA. Parvizi et al. found that 49.4% of hip OA patients had severe LBP, with 66.4% of them experiencing postoperative resolution [7]. Chimenti et al. reported that 35.3% had moderate to severe LBP before THA, with 55.8% seeing their LBP reduced to either mild or none afterward [8]. Piazzolla et al. found severe LBP in 51.6% of hip OA patients, with the VAS score decreasing from 5.3 to 1.07 postoperatively [9]. The Hsieh et al. study showed that 21.2% of hip OA patients had LBP, with the VAS score dropping from 3.7 to 0.0 six months postoperatively [10]. In a study by Ben-Galim et al., all 25 THA patients had at least moderate LBP preoperatively, which decreased from 5.04 to 3.68 and 3.64 at three- and 24-month postoperative check-ups, respectively [11]. Staibano et al. also reported that 28.8% of patients with hip OA and LBP saw a 53.7% improvement one year postoperatively [12]. Okuzu et al. reported a postoperative improvement in 61.7% of patients who experienced LBP before THA [13]. Saiki et al. suggested that 37% of THA patients had postoperative LBP, with 54% seeing pain improvement [14]. Ran et al. indicated significant improvement, denoted by the decrease in the VAS score for LBP from 4.18 preoperatively to 1.95 at 44 months after the surgery [15].

International literature presents only a few studies outlining theories that suggest a correlation between LBP and hip OA in patients who have undergone THA. Essentially, two primary theories are proposed. The first theory is that LBP in these patients originates from abnormal spinal, pelvic, and hip parameters. The reduction of LBP post-THA is attributable to the subsequent correction of these parameters. The second theory asserts that the alleviation of LBP post-surgery results from decreased hip pain and improved functionality and range of motion in the hip following THA.

Multiple studies substantiate the initial theory. The first shows that hip OA contributes to anterior pelvic tilt, compensatory hyperlordosis of the lumbar spine, and, eventually, LBP caused by subluxation or overloading of the posterior articular processes [1]. The second study suggests hip OA leads to lumbar scoliosis, and its significant postoperative reduction improves LBP [16]. The third study indicates a strong correlation between a higher femoral neck anteversion angle in the osteoarthritic hip joint and LBP. Correcting this angle postoperatively can potentially alleviate LBP by improving trunk parameters and fixing the antalgic gait [9]. The fourth study posits that preoperative moderate to severe pelvic retroversion is significantly linked to postoperative LBP [17]. The fifth study finds patients with preoperative variations in sacral slopes from sitting to standing over 10 degrees are more likely to achieve LBP resolution. Consequently, the rigidity of the sacro-pelvic complex might play a role in persisting LBP after THA [5]. Lastly, the sixth study shows significant variances in spinal parameter changes in the LPB-improved group, which includes heightened lumbar lordosis (LL), reduced sagittal vertical axis (SVA), and pelvic incidence (PI) minus LL postoperatively. In contrast, the LBP continued group displayed disimproved changes in LL, SVA, and PI-LL mismatch [13].

Several studies affirm the second theory. Tanaka et al. demonstrated a correlation between the range of motion in hip flexion on the affected side and LBP in patients with hip OA [18]. Ben-Galim et al. found that improvements in hip function and reductions in hip pain three months post-surgery corresponded closely with LBP reduction [11]. During a follow-up two years after the operation, even greater LBP reductions and improved spinal function were observed alongside continued advancements in hip functionality. Redmond et al.'s systematic review corroborated this theory, underscoring a connection between postoperative improvements in hip joint pain and range of motion and LBP reduction [19]. Chimenti et al. reported that patients who experienced LBP alleviation also saw significant pain reduction in both the operated and unoperated hips [8].

Weng suggests that postoperative improvement in overall spinal balance parameters and hip flexion can contribute to the alleviation of LBP. This can be achieved by reducing back muscle tension, enhancing the range of hip motion, and lessening stress in the lumbopelvic area [6]. Weng et al. believe that spine morphology and hip function could both be contributing factors to LBP [6]; thus, combining the two main theories is recommended. However, no significant difference was found in sagittal alignment between patients with and without LBP one year post-surgery. Both patient groups exhibited similar changes in hip flexion and restoration of spinal balance following THA.

Similar studies, such as those by Weng et al. [6], have demonstrated that patients with hip OA do not show statistically significant improvements in sagittal and coronal alignment of the spine and pelvis, even if LBP improves following THA [14,20]. Therefore, it is suggested that the mechanisms relieving LBP may be more complex and not yet fully understood.

This study found that 79% of patients who had LBP prior to THA noted an improvement post-surgery. The only other improved components were hip pain and pelvic obliquity. We analyzed the correlation between LBP and these parameters. Both sets of patients, those with and without preliminary LBP, experienced identical changes in hip pain and pelvic obliquity after undergoing THA. However, no clear link between LBP and hip pain or pelvic obliquity was established in this study. Nonetheless, this conclusion does not eliminate the possibility of such a correlation. A larger patient sample could potentially reveal a statistical correlation between these factors. We suggest that the cause of LBP is complex and may be associated with hip pain or pelvic obliquity.

Knee OA, TKA, and LBP

In our prospective study, we observed that 62.22% of patients with severe knee OA who underwent TKA experienced moderate to severe LBP. These patients significantly decreased their VAS score for LBP from 6.39 preoperatively to 4.79 at four months postoperatively and further to 4.04 after 12 months. Moreover, the LBP score decreased in 50% of the patients, and 28.6% notably reduced their LBP score by more than four units 12 months after the operation.

These results align with those from similar studies. Kitagawa et al. found that 66% of knee OA patients experienced LBP, with a third reporting decreased LBP one year after TKA [21]. Similarly, Staibano et al. indicated a 16.1% prevalence of LBP among knee OA patients, with roughly 31.6% noting improvements postoperation [12]. In a study by Collados-Maestre et al., only four out of 48 knee OA patients with preoperative LBP reported improvements three years after TKA [22]. According to Ayers et al., 52.6% of knee OA patients reported LBP at the time of surgery, while Suri et al. found that 57.4% of knee OA patients experienced LBP [23,24]. Wolfe et al. reported that 54.6% of knee OA patients suffered from LBP [2]. Burnett et al. discovered that 74% of knee OA patients reported chronic LBP, which first occurred roughly 10 years prior, and less than 15% claimed their worst LBP began after the onset of knee OA [25]. Furthermore, Iijima et al. highlighted that 58.1% of knee OA patients undergoing TKA experienced LBP [26].

The international literature presents just three studies exploring the prevalence of LPB in patients with knee OA. Only one study identified a correlation between the reduction of LBP post-TKA. Similar to hip OA, two primary theories strive to explain the correlation to the KSS. The first contends that knee and spine morphological abnormalities cause the LBP in these patients. The second asserts that intense knee pain resulting from OA generates the LBP.

Studies exist that uphold the initial theory. One such study suggests that degenerative changes occurring in knee OA can lead to a loss of knee extension, subsequently causing a reduction in LL [27]. Increased intradiscal pressure and disc degeneration, associated with reduced LL, could potentially lead to LBP. Similarly, Harato et al. affirmed that a knee flexion contracture exceeding 30 degrees can alter trunk kinematics across all planes, which may correlate with LBP [28]. However, it is worth noting that none of the studies mentioned included the measurement of the LBP parameter.

A study indicates that LBP correlates with elevated knee pain scores in OA patients. This pain linkage exists because knee pain and function impact adjacent joints along the kinetic chain [24].

Kitagawa et al. demonstrated that patients with knee OA and LBP often have a forward tilt in their overall sagittal alignment. However, this imbalance was not corrected after TKA, and there was no noticeable difference in postoperative spinopelvic parameters among patients, regardless of whether their LBP improved or not. Therefore, it appears that there's no connection between sagittal global alignment and LBP in patients with knee OA [21].

This study revealed that post-TKA, 50% of patients with preoperative LBP saw improvements. There was also an improvement in knee pain and pelvic obliquity. We aimed to assess any correlation between LBP, knee pain, and pelvic obliquity. Upon comparison, patients with and without preoperative LBP showed similar changes in knee pain and pelvic obliquity post-TKA. Consequently, we could not establish a clear correlation between LBP and knee pain or pelvic obliquity, albeit not completely ruling out potential correlations. We anticipate that a larger patient sample might reveal a statistically significant relationship between these variables. We propose that the causes of LBP are likely multifaceted without discarding the possibility of a link between knee pain, pelvic obliquity, and LBP.

Comparison of LBP between patients with hip OA and knee OA

Our study interestingly compares LBP in patients with hip and knee OA. LBP prevalence is higher in patients with knee OA than in those with hip OA (62.22% vs. 55.88%). Notably, LBP improvements are more significant in hip OA patients (6.84 to 2.58 (four months postoperative) and then to 2.53 (12 months postoperative)) compared to knee OA patients (6.39 to 4.79 (four months postoperative) and then to 4.04 (12 months postoperative)). More patients with hip OA, as compared to those with knee OA, demonstrated a significant LBP reduction of over four units (47.4% vs. 28.6%). Thus, the greater decrease in LBP VAS scores in patients undergoing THA compared to those undergoing TKA might be due to the hip's closer proximity to the spine than the knee.

Only two studies have made comparisons between patients suffering from hip and knee OA. Stupar et al. found a stronger correlation between LBP, joint pain, and disability in patients with hip OA compared to knee OA, contrary to our research [29]. This divergence could be attributed to more severe knee flexion contractures in knee OA patients included in this study. Severe unilateral knee flexion contracture causes a higher functional leg length discrepancy and possibly worse LBP due to trunk imbalance.

On the other hand, Staibano et al. reported that the prevalence of significant preoperative LBP was considerably higher in hip patients at 28.8%, compared to 16.1% of knee patients, which contrasts with our findings. Moreover, in the same study, 53.7% of hip OA patients and 31.6% of knee OA patients experienced improvement one year after surgery. These results align with our findings [12].

Limitations

This study has some limitations. Firstly, the small sample size is a constraint despite ultimately yielding statistically significant results. Secondly, the follow-up period is short and could be improved with a more extended duration.

## Conclusions

HSS and KSS are prevalent diseases that significantly affect quality of life. Our study confirms this frequency and notes an improvement in LBP in hip or knee OA patients following THA or TKA. Consequently, we propose that total arthroplasty should precede spinal surgery in patients suffering from joint arthritis and LBP. Importantly, LBP should not be viewed as a contraindication for THA or TKA. Our findings suggest that hip or knee pain and pelvic obliquity were the primary parameters that improved post-THA or TKA, excluding LBP. However, no evident link between these two parameters and LBP was established. Nevertheless, this situation seems complex, and further studies are needed for clarification.
